# An Extensive Study of Convolutional Neural Networks: Applications in Computer Vision for Improved Robotics Perceptions

**DOI:** 10.3390/s25041033

**Published:** 2025-02-09

**Authors:** Ravi Raj, Andrzej Kos

**Affiliations:** 1 Institute of Robotics and Machine Intelligence, Poznań University of Technology, ul. Piotrowo 3A, 60-965 Poznań, Poland; 2 Faculty of Computer Science, Electronics, and Telecommunications, AGH University of Krakow, al. Adama Mickiewicza 30, 30-059 Kraków, Poland; kos@agh.edu.pl

**Keywords:** artificial intelligence (AI), computer vision, convolutional neural network (CNN), deep learning (DL), machine learning (ML), mobile robot (MR), perception

## Abstract

Convolutional neural networks (CNNs), a type of artificial neural network (ANN) in the deep learning (DL) domain, have gained popularity in several computer vision applications and are attracting research in other fields, including robotic perception. CNNs are developed to autonomously and effectively acquire spatial patterns of characteristics using backpropagation, leveraging an array of elements, including convolutional layers, pooling layers, and fully connected layers. Current reviews predominantly emphasize CNNs’ applications in various contexts, neglecting a comprehensive perspective on CNNs and failing to address certain recently presented new ideas, including robotic perception. This review paper presents an overview of the fundamental principles of CNNs and their applications in diverse computer vision tasks for robotic perception while addressing the corresponding challenges and future prospects for the domain of computer vision in improved robotic perception. This paper addresses the history, basic concepts, working principles, applications, and the most important components of CNNs. Understanding the concepts, benefits, and constraints associated with CNNs is crucial for exploiting their possibilities in robotic perception, with the aim of enhancing robotic performance and intelligence.

## 1. Introduction

Convolutional neural networks, or CNNs, have been achieving incredible results. It is now among the most prominent artificial neural networks (ANNs) in the context of deep learning (DL). CNN-based computer vision has made it feasible for people to perform tasks that were thought to be unthinkable just a few generations ago, including self-service grocery stores, human activity recognition, driverless cars, facial recognition, robotic perception, and smart healthcare [1,2]. CNNs’ ability to reduce the quantity of ANN variables is their greatest advantage. The primary presumption regarding CNN-solved issues is that they must not include spatially dependent elements. Extracting abstract characteristics as input progresses toward the deeper layers is another crucial component of CNNs [3]. CNNs’ primary advantage over their predecessors lies in the way they can automatically identify relevant information without supervision from humans [4]. In this article, we discuss the fundamental framework of CNNs, describe traditional models and applications in computer vision for robotic perception, and suggest possible future paths for CNNs with the goal of gaining a better comprehension of modern CNNs and rendering them more useful to humans for effective robotic perception.

**Figure 1:**
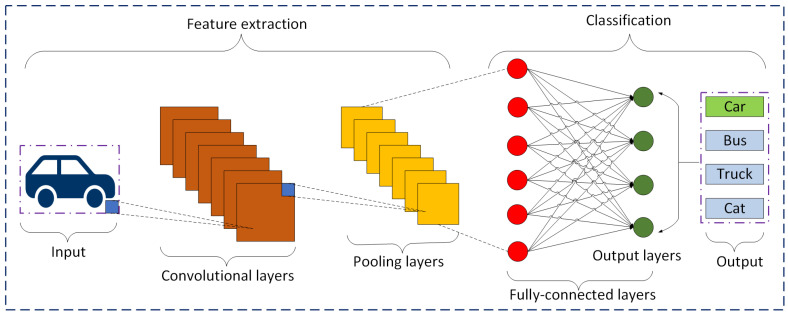
Illustration of the basic architecture of a CNN-based image classification technique.

CNNs are a standardized variant of a feed-forward neural network that autonomously learn features through kernel optimization. The architecture of CNNs was influenced by the neurons in animal and human brains and is compatible with traditional neural networks [5]. CNNs have undergone continuous evolution and refinement. The initial development was made in the late 1980s with the creation of LeNet by Yann LeCun. LeNet established the structure to obtain CNNs and was mostly utilized for recognizing digit jobs [6]. CNNs classify images and recognize objects using three-dimensional input. Before CNNs, entities from photos were identified using difficult, traditional feature extraction techniques. However, by using concepts from linear algebra, particularly the use of matrix multiplication to find patterns in a photograph, CNNs currently offer an additional scalable method for image and object recognition applications. CNNs outperform different types of neural networks when the given input is in audio, speech, or images [7]. A CNN consists mainly of three layers including convolutional layers, pooling layers, and fully connected layers. Figure 1 shows an illustration of the basic architecture of a CNN. When an image of a car is taken as input for a CNN-based image classification system, it can be easily recognized as an image of a car, which is shown by a green-colored block.

Given that robots are entering our daily lives via automated industries, it makes sense to provide them with certain previously indicated abilities (such as several social norms) that are focused on the difficulty of dealing with humans. It is essential in this situation to possess robotics perception technology that can interpret the social signals that are revealed throughout interactions [8]. The goal is to anticipate the actions taken by humans within the robotics environment and to create a socially acceptable and seamless connection between robot and human. Being able to predict the upcoming actions and motivations of those around us can assist the robot in predicting its actions and responses. Robots must be able to interpret both verbal and nonverbal communication signals from every human being, as well as the dynamics in relationships with others [9]. Body activity, facial expressions, and gestures are all important indicators of a human’s inner condition. The robot can demonstrate appropriate conduct from the perspective of society by comprehending the complexities of a community of humans and determining all of its social significance. Only through human observation, modeling, and interaction with others can every aspect of this information be learned by robots.

**Figure 2:**
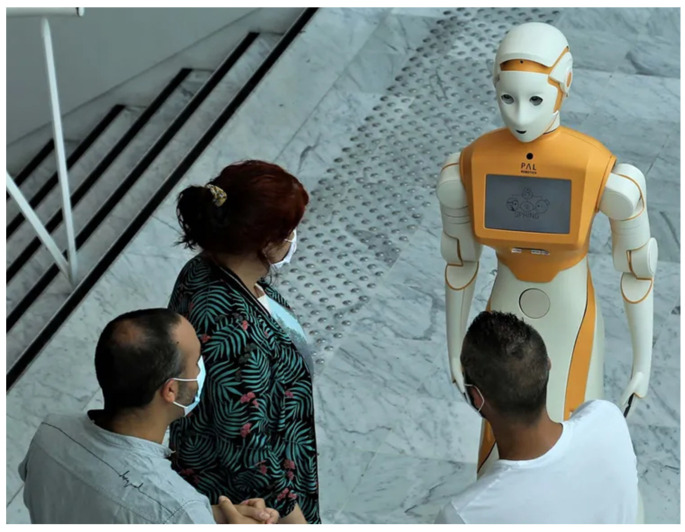
Illustration of an interaction between social robots and patients and caregivers [10].

In recent years, social robots have been successfully deployed in public venues, including libraries, airports, shopping malls, financial institutions, corporate showrooms, healthcare facilities, and elderly care facilities, among others. Besides traditional robotic competencies, including transportation, grasping, and object manipulation, social robots must additionally have the skill to interact with humans in a relatively natural manner, for example, cognitive interactions. To effectively perform social duties and accomplish social activities, it is essential for robots to possess the capabilities to navigate, perceive, cognitively interpret, and interact within complex and unorganized environments crowded with people. Figure 2 shows an illustration of a social robot interacting with patients and caregivers. Thus, it is necessary for social robots to become more autonomous. For the betterment of social robots’ adaptability, this study provides a deeper study into the process of robotic perception.

A CNN framework is a type of DL algorithm that uses large amounts of information to assess and learn features. The technique of creating and implementing a CNN framework involves three steps: inference, training, and optimization. Developments in computer vision through DL have been developed and refined over time, mostly centered around one specific technique known as CNN. Computer vision speeds up image processing and analysis by combining software and hardware that have been designed for CNN functioning. Traditional neural networks are reassembled by CNNs, but it has a particularly interesting feature: neurons containing learnable biases and weights [11]. After receiving several inputs, each neuron executes a dot product that can or cannot be preceded by nonlinearity. Consequently, the CNN undergoes several adjustments and operates as a feed-forward kernel [12]. The design of CNNs parallels the connection network of neurons in the human brain, and CNNs are inspired by the structure of the visual cortex. Only in a limited area of the field of vision described by the receptive field do specific neurons react to inputs. The whole visual region is shaded with an overlap of these fields [13]. Convolutional networks are the prevailing norm in deep DL methodologies for computer vision and image analysis and have just recently been supplanted—in specific instances—by novel DL structures like the transformer. Major parts of CNNs are described in the following ways:

### 1.1. Convolutional Layers

**Figure 3:**
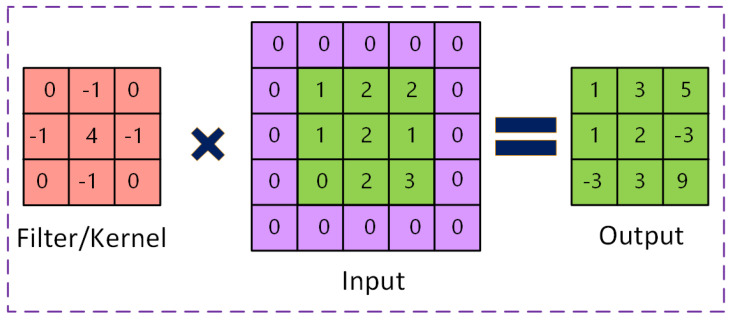
Illustration of an example of the convolution operation.

A convolutional layer serves as the fundamental component of CNNs. It comprises a collection of kernels (or filters), which are the parameters that must be acquired during the training process [14]. The dimensions of the filters are often smaller than those of the original image. Each kernel convolves with the photo to generate an activation map. During convolution, the kernel traverses the height and breadth of the picture, computing the dot product between each component of the kernel and the data provided at each spatial location [15]. Figure 2 illustrates an instance of the convolution operation. In Figure 3, the filter is convolved with the green area of the input picture to determine the initial entry of the activation map (shown in green). This procedure is repeated for each component of the input picture to create the activation map. The activation maps of each filter are stacked along the depth dimension to create the convolutional layer output volume. One might believe that each element of the activation map represents the result of a neuron. As a result, each neuron is linked to a tiny, localized region of the input picture, and the size of that area is the same as the filter’s [16]. For an activation map, every neuron shares parameters with each subsequent neuron. The network must train filters with the highest response to a specific area of the input because of the convolutional layer’s regional connectivity. The lower-level attributes of images are captured by the first convolutional layers, whereas higher-level attributes are extracted by the subsequent layers.

### 1.2. Pooling Layers

A stochastic technique called pooling allows outputs at a specific place to be combined into a single value. This distinctive value is derived from the statistical analysis of successive outputs, enhancing the precision and sensitivity of feature interpretation for reduced input datasets [17,18]. Information scattered over several vectors is downsampled and aggregated into smaller vectors using a type of CNN layer known as a pooling layer. The pooling layer progressively reduces the dimensions of the input, thereby lowering memory use to retain parameters and enhance statistical performance [19]. Pooling layers are employed in neural networks to mitigate overfitting [20].

**Figure 4:**
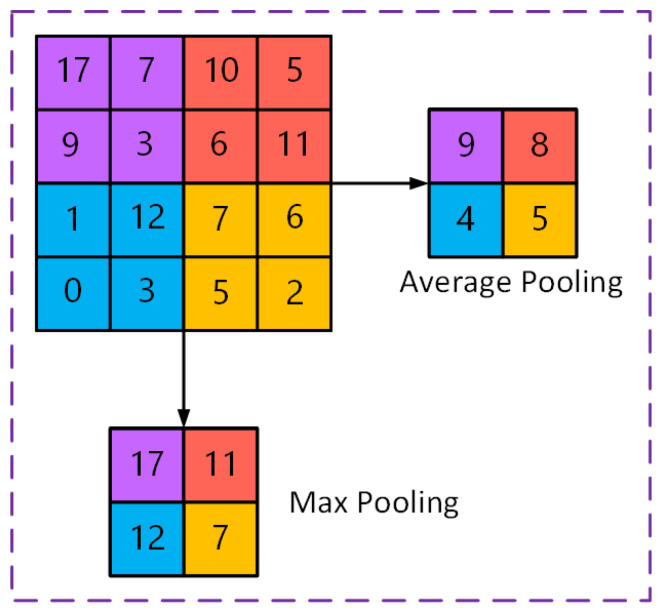
Illustration of an example of average and max pooling.

Pooling is a crucial stage in convolution-based techniques for lowering the size of features that are gathered. The feature map’s sizes are minimized by lowering the quantity of parameters in the numerical sequence. By keeping the material pertinent and eliminating unnecessary data, it transforms basic descriptions of features into information that can be used [21]. The pooling process lowers the computing expenses of the upper layers and gives additional spatially modified information by eliminating the incomplete connectivity that exists between convolution layers. It creates numerous feature maps with constrained resolution by sampling features that have been obtained from the preceding layer. Figure 4 shows an example of average and max pooling calculations. The max pooling method finds every pooling region’s largest component, and average pooling is calculated by averaging all components in each pooling region. The mathematical expression for average pooling is as follows, in Equation (1) [21]:

(1) $$P_{avg}=\frac{1}{N}\sum_{i}^{N}\left(y_{i}\right)$$

where, the vector y represents the activations for a rectangular frame of N permutations within an image.

The mathematical expression for max pooling is as follows, in Equation (2) [21]:

(2) $$P_{max}(Y)={max}_{i}y_{i}$$

### 1.3. Fully Connected Layers

Basically, fully connected layers are feed-forward neural networks. The final few network levels are called fully connected layers. The output of the last pooling layer is fed into the fully connected layer after being flattened. Some of the fully connected layers that function similarly to ANNs and carry out similar mathematical computations are subsequently connected to this flattened vector [22]. Each ANN layer undergoes the subsequent computation, as shown in Equation (3) [22]:

(3) f(Wz+b)

where *f* is an activation function, *W* is the weight matrix, *z* is the input vector, and *b* is the bias vector.

CNNs greatly improve the vision and decision-making skills of robots. This study is dedicated to exploring the basic concepts, recent techniques, and relevant aspects addressing humans and environmental perception for enhancing CNN-based robotic perception. The acquisition of this highly complicated expertise, limited to recognizing signals enabling social perceptions, demands that a robot comes equipped with both hardware and software components that enable it to (i) recognize humans and their respective positions, either static or dynamic, and (ii) associate the newly acquired features to a particular, learned, or online gathered state (social information) to perform its modeling. This study reviews DL techniques to analyze complicated settings by investigating several computer vision approaches, and it investigates the application of CNNs to enhance the reliability of object and human gesture classification. In short, the main contributions of this paper are as follows: (i) We provide a comprehensive introduction to CNN and its components in detail for robotic perception applications. (ii) We explain the application of various human activities and intention recognition for perception applications. (iii) We provide a deeper analysis of challenges and future research directions in the field of robotic perception.

The remaining part of this article is divided into four parts: Section 2 explains the major types of CNN; Section 3 provides a survey of the most recent related articles; Section 4 presents some major applications of CNNs for robotic perception; Section 5 provides CNN-based robotic perception; Section 6 contains information about the future research trends and challenges; and lastly, Section 7 provides concluding remarks.

## 2. Types of Convolutional Neural Networks

**Figure 5:**
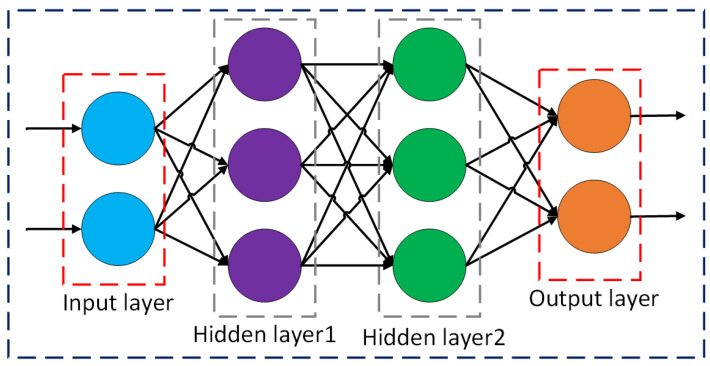
Basic structure of a convolutional neural network.

CNNs have transformed computer vision by enabling improvements in object identification, picture recognition, and other visual applications. CNNs’ capacity to automatically extract structural representations from data is what makes them so successful. The broad range of CNN types illustrates how DL has changed over time [23]. The advancement of large-scale computation and GPU acceleration has made it possible to create massive CNN frameworks comprising several convolutional layers. Robots will probably need a better grasp of the environment that works for the purpose of operating and connecting with it in greater depth. CNNs are widely used nowadays to perceive environments for the navigation of robots. CNNs’ architecture is growing increasingly sophisticated and diverse as a result of the quick advancements in DL technology. As a result, it progressively supplants conventional ML techniques. Figure 5 provides a basic structure of a classical CNN.

**Figure 6:**
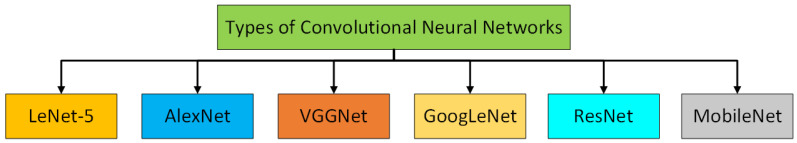
Illustration of major types of convolutional neural networks.

CNN-based action detection has been effectively expanded from visual to deep and thermal video, as well as from single-view to multi-view vision. Classification, a fundamental and vital activity that has been significantly explored, is always included in the detection task. Identifying and comprehending human behaviors and intents is crucial in robotic perception, HRI, intelligent monitoring, and related fields, making it a significant and prominent area of research [24]. CNNs are a type of DL model that are primarily developed for processing image and video datasets. Different types of CNN models are developed, each of which is used for specific problems and types of complexities. Some major types of CNNs based on their architecture are illustrated in Figure 6.

### 2.1. LeNet-5

LeNet-5, which has a very basic design, is often considered the first CNN. The most traditional usage of LeNet, for which it was designed, is the recognition of images with fundamental numbers [6]. In contrast to conventional techniques, LeNet-5 presented a revolutionary method that processed pixel pictures immediately using convolutional layers, subsampling, and fully connected layers, removing the requirement for human feature engineering. It consists of only seven layers, and out of seven, three are convolutional layers, which are interconnected with two pooling layers. The remaining layer is a fully connected layer, and one layer is the output layer, respectively. For effective learning, it employs a sequence of layers that acquire unique characteristics from the data and normalize the input pixels. Its popularity went beyond character recognition; it influenced later designs in computer vision, object identification, and picture classification, and it formed the basis for contemporary DL models [25]. LeNet-5 was created in 1995, and several techniques for handwritten character recognition were examined and contrasted with industry-standard standards for handwritten digit recognition.

### 2.2. AlexNet

AlexNet refers to a convolutional neural network architecture developed by Alex Krizhevsky in conjunction with Ilya Sutskever and Geoffrey Hinton, who served as Krizhevsky’s Ph.D. advisor at the University of Toronto. It possessed 60 million variables and 650,000 neurons [26]. The preliminary paper’s principal finding proved that the model’s complexity was crucial for its superior performance, which was extremely computational but became possible with the use of graphics processing units (GPUs) for training [26]. The design significantly impacted several subsequent developments in deep learning, particularly in the application of ANNs to computer vision.

### 2.3. VGGNet

VGGNet is a prototypical deep CNN architecture characterized by its extensive layering, with the acronym VGG standing for Visual Geometry Group. The object detection technique designed and trained by Oxford’s esteemed VGG, known as VGGNet, is far superior to the ImageNet dataset [27]. Furthermore, it remains one of the most widely used image recognition frameworks today. Innovative object recognition techniques are based on the VGG architecture. The VGGNet is built using extremely small convolutional filters. Since many modern image classification techniques are built on a foundation of VGGNet, it is crucial to understand it.

### 2.4. GoogLeNet

One type of CNN that is based on the Inception design is called GoogLeNet. In 2014, Google researchers introduced Inception, a CNN family for computer vision, as GoogLeNet [28]. Through the application of Inception modules, the network is able to select among a variety of convolutional filter dimensions for each block. These modules are stacked one on top of the other in an Inception network, with stride-two max-pooling layers added occasionally to cut the grid’s resolution in half. As a pioneering CNN that divides the stem (data intake), body (data processing), and head (forecasting), the series was significant strategically. This architectural design is still used in all contemporary CNNs.

### 2.5. ResNet

Residual Neural Networks (ResNets) are a common type of CNN that improves obtaining information from input data and successfully solves the loss of information issue [29]. It accomplishes this by using an identity mapping program that includes alternatives or bypassing links to traverse network tiers. ResNet can now reach an unprecedented 152 layers deep. Rather than learning unreferenced functions, ResNet formally reformulates the layers as learning residual functions according to the layer input [26]. According to thorough empirical data shown by He et al. [30], ResNets are simpler to tune and can improve accuracy with significantly greater depth.

### 2.6. MobileNet

MobileNet is a unique type of CNN, a computer vision model created for classifier training and made publicly available by Google. It creates a compact deep neural network (DNN) by drastically reducing the quantity of parameters when compared with conventional networks through the incorporation of depth-wise convolutions. It is considered to be the first computer vision model for TensorFlow [31]. The quantity of multiply-accumulates, an indicator of the quantity of fused addition and multiplicative activities, is directly correlated with the speed of the network and energy consumption. Howard et al. [32] present two fundamental global hyperparameters that effectively balance accuracy and latency. Based on the limitations of the issue, the model developer can select the appropriate model dimension for the application using these hyperparameters.

## 3. Related Work

Recently, many researchers are working towards developing an efficient CNN-based approach in computer vision for robotics perception. Cereda et al. [33] propose using a vision-based perception methodology in non-egocentric mediated activities, where the predicted outputs pertain to an external topic. This study demonstrates how the proposed approach’s overarching technique enhances the regression efficacy of deep CNNs across a wide range of non-egocentric 3D pose estimation challenges while incurring minimal computational expense. This work validates the in-field effectiveness of a closed-loop automated centimeter-scale UAV with the human pose estimation trial.

Aulia et al. [34] present a novel CNN-based object recognition system for an autonomous mobile robot utilizing real-world vehicular datasets. A unique real-world dataset of photographs from Banda Aceh City was compiled. A novel CNN-based object recognition system was created, which is proficient in distinguishing vehicles, motorbikes, individuals, and rickshaws throughout early morning, midday, and sunset illumination situations. The suggested CNN-based object recognition system possesses the capability for application in an autonomous mobile robot (AMR).

Foroughi et al. [35] propose a system that enables the mobile robot to automatically locate and move within the traversable regions of an interior environment. In an effort to accurately identify the position zone of the mobile robot by concentrating on the geometric maps of the actual environment, the suggested system takes advantage of the CNN system’s ability to acquire information from map features, enabling the classification of images and visual localization. By combining the CNN model with the geometrical map for the robotics work area, the system aims to accurately identify the position zone of the mobile robot. According to the results, the suggested system performs better in terms of reliability and generalization capacity than other cutting-edge methods. The suggested loss function takes into account both the likelihood that the input data will be assigned to its true class and the likelihood that it will be assigned to different classes instead of its true class.

Hoshino et al. [36] present a CNN-based motion planner with long short-term memory (LSTM) enabling the avoidance of obstacles using mediated perception. When it deals with avoiding obstacles, the robot must plan avoidance maneuvers while taking into account the time-series fluctuation in the pictures, since the orientation of a dynamic obstacle varies over time. This can be done by applying an LSTM block to the CNN. Through the use of LSTM along with CNNs, the motion planner’s policies receive instruction via imitation learning. A perception method is additionally supplied between the picture data and the CNN with LSTM within the motion planner, allowing obstacle identification. With the help of the recommended motion planner, the robot can navigate independently toward its target while avoiding both walking and standing people.

Chen et al. [37] provide a paradigm for tactile perception that combines explicit and latent connection graphs and depends on graph attention networks. This system might make good use of the structural data that exists between various tactile signal channels. The graph-based tactile perception method is more appropriate for interpreting multi-channel tactile data than current time-series signal classification techniques because it can more effectively use and acquire sensor spatial information. This technique might be used as a general approach to enhance a robot’s tactile perception.

Alazeb et al. [38] present a multi-object recognition system leveraging remote smart perception. The fields of multiple object recognition and visual interpretation are significantly impacted by developments in vision technology. These activities are essential components of many technologies, including virtual reality scenario integration, robot navigation, self-driving vehicles, and enhanced tourist guidance apps. Although visual interpretation has made great progress, many issues remain, including semantic comprehension, orientation, occlusion, lack of labeled information, inconsistent illumination (darkness and light), direction change, object dimension, and shifting background. This approach offers a novel scene recognition architecture to address these issues, and it has proven to be quite successful in producing impressive outcomes.

Momen et al. [39] examine if perceivers’ more advanced views regarding faces (for instance, if they represent real individuals or androids) influence the degree to which observers utilize face-typical reasoning for social cues. Previous research indicates that the identification ability of observers is more adversely affected by the reversal of faces compared to objects, underscoring that faces are interpreted comprehensively (i.e., as a Gestalt); however, objects are analyzed primarily based on individual features. This study used an inversion experiment to investigate whether face-typical cognition diminishes when genuine human faces are categorized as not human (i.e., artificial robots). The findings indicate fewer inversion implications for face stimuli perceived as android robots in contrast to those perceived as humans. This indicates that humanoid robots might continue to be viewed as non-social due to entrenched ideas regarding their mechanical essence.

Ran et al. [40] propose a lightweight robotic navigation system utilizing uncalibrated spherical pictures. The navigation issue has been split into a number of categorization tasks in order to streamline orientation estimations, path predictions, and increase computing performance. This work presents the spherical camera for picture collecting, which allows 360° fisheye panoramas as samples for training and the creation of adequate positive as well as negative traveling directions, for the purpose of minimizing the detrimental impacts of inadequate negative data during the “navigation via classification” assignment. The suggested Spherical-Navi visual dataset, where category labeling might be easily gathered, is used to train the end-to-end CNN used for classification. With only an uncalibrated spherical picture, the CNN model can make very confident predictions about possible travel orientations.

Fu et al. [41] present a generative robotics grasping technique based on CNN. For intelligent robots to execute picking jobs, grasp planning for unplanned environments with optimal performance and effectiveness is a crucial issue that must be resolved immediately. A generative neural network with regulated grasp efficiency has been proposed as a solution to this issue with the objective of creating pixel-level grasps. An adaptive filtering technique is suggested to filter the grasp designs with the aim of increasing the model’s generalization and resilience. To determine the last grasp arrangement, an elliptical fitting-based grasp posture optimization strategy is then presented. The model’s viability has been confirmed by deploying it on embedded AI computational equipment.

## 4. Major Applications of CNNs for Computer Vision

Computer vision techniques are used in industries, including healthcare, military surveillance, and industry, to acquire information from images. The rapid advancement of CNNs has led to a variety of significant improvements in computer vision, including recognizing objects, segmenting semantically, picture classification, and high-resolution image reconstruction. The autonomous robot’s ability to do its duty with minimal or no help from humans is crucial. Autonomous robots need no oversight; they can operate alongside or replace people to execute jobs that humans are unable to, ought to avoid, or prefer not to undertake, particularly in hazardous environments and locations with rapid disease dissemination [42,43]. Self-learning robots have been utilized across several sectors and purposes, including production, agriculture, warehouse operation systems, healthcare sectors, and military operations. Despite the many potential uses of autonomous robots, such robots need a diverse array of control techniques and capabilities to execute their intended functions efficiently [44]. One of these is to use object recognition or sensor perception to comprehend their surroundings and navigate free of obstacles. Reinforcement learning [45] and DL techniques like K-Nearest Neighbors (KNN) [46] and CNNs are frequently employed in sensor perception to interpret sensor input and assist the robot in sensing its operative environment.

**Figure 7:**
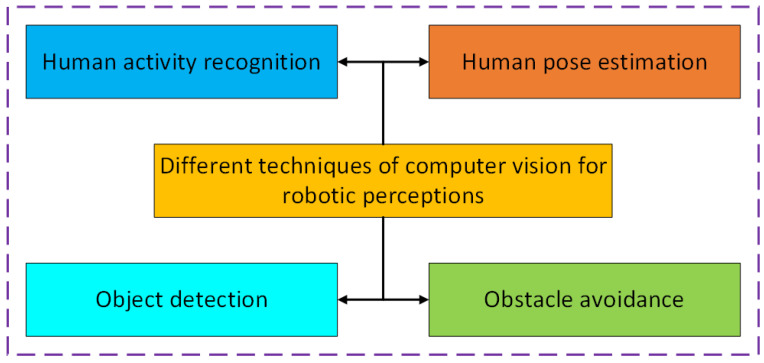
Illustration of different techniques of computer vision for robotic perception.

Autonomous robots can detect their environments by employing computer vision techniques, including human activity recognition (HAR), human pose estimation, and object recognition in addition to sensor perception [47]. CNNs are the most widely used deep learning method in computer vision for the optimization of robotic perception toward intelligent motion planning for robots. It offers a prospective capacity in obstacle avoidance scenarios by employing computer vision-based obstacle detection to guarantee collision-free operations for autonomous robots [48]. Rather than processing input from sensors, a CNN, an approach for classification, might be employed for training a vision-based system for avoiding obstacles, utilizing real-time images acquired by a camera. CNNs can be widely used for various types of computer vision techniques, including HAR, human pose estimation, object detection, and obstacle avoidance, which are crucial to enhanced robotic perception. Computer vision with CNNs empowers robots to comprehend data from their environment, enhancing object detection, navigation, and interaction with their surroundings. Some important computer vision with CNN-based techniques that can be useful for the robotic perception are described in Figure 7. These computer vision-based perceptual skills allow robots to traverse intricate landscapes, circumvent obstacles, and provide educated judgments based on visual context.

### 4.1. Human Activity Recognition

**Figure 8:**
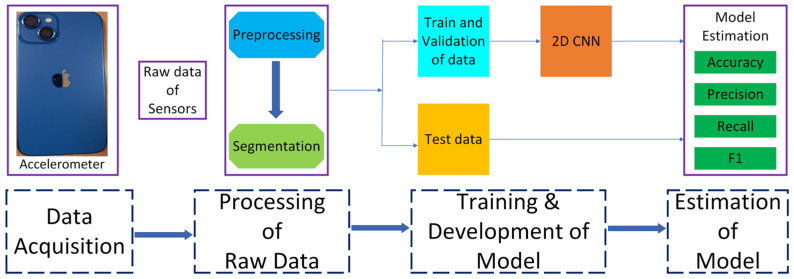
An illustration of the human activity recognition framework using CNNs [53].

The task of categorizing human everyday behaviors can be referred to as human activity recognition (HAR). HAR represents one of the primary applications of wearable devices for healthcare surveillance, which necessitates continuous monitoring of daily activities [49]. Robots rely heavily on perception in order to build a representation of both their internal and external environments. Perception is essential to robotics because it allows robots to interact with humans and the environment in a natural way, improving user experience, efficiency, and safety [50]. In developed nations, HAR is unavoidable for consistent human–robot interaction (HRI) because it supports a growing number of mobile robots, including assistive robots for healthcare support and the assistance of persons with disabilities, and household robots for maintenance and cleaning. Autonomous robots can navigate in unpredictable surroundings without assistance by quickly learning human behaviors when they are equipped with the HAR technique [51]. Recently, many studies [52,53,54] have been conducted to perform HAR using CNNs for improved robotic perception. Figure 8 describes a complete framework for HAR using CNNs. The HAR framework starts with data acquisition using an accelerometer sensor and ends with model estimation by finding accuracy, precision, recall, and F1 scores of the experimental tasks.

### 4.2. Human Pose Estimation

**Figure 9:**
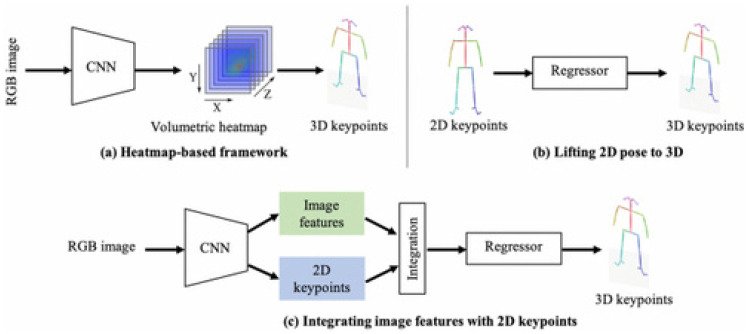
An illustration of the human pose estimation framework using CNNs [59].

Human pose estimation is a significant and difficult computer vision topic that has been addressed by several techniques. Interaction between humans and robots can occur in various ways. One way to do this is via physical gestures, such as pointing to an item or issuing commands. After that, the robot ought to be capable of reading the gestures and responding appropriately to the person in a real-world situation. Service robots, intelligent systems that combine many perceptual modalities, are growing in demand in the modern world as a result of the ongoing advancements in technology [55]. These robots can operate exceptionally well across a variety of challenging activities and locations because it is possible to not only collect and analyze visual input but also integrate data collected by additional sensors, including force and sound. Some of the most important challenges in enhancing the perception and cognitive skills of service robotics are human pose estimation. Accurate information regarding human body position and mobility is crucial for successful HRI and health tracking, among other activities. CNNs play an important role in human pose estimation. Recently, various studies [56,57,58] have shown an enhanced human pose estimation technique using CNNs. Figure 9 provides an illustration of human pose estimation frameworks using CNNs. Human pose estimation frameworks start with the acquisition of the input image in the form of an RGB image and end with each major keypoint detection in the human skeleton, which represents the corresponding pose of the human skeleton in the input image.

### 4.3. Object Detection

In computer vision, object recognition is a challenging problem that involves recognizing and categorizing items in a picture or video. The ability of CNNs to extract complex characteristics and patterns from unprocessed image data has made them the most widely used and successful method for object recognition at present. In robotics, recognizing objects is essential for allowing robots to properly perceive and interact with the environment. Robots may use cameras to recognize items in their environment with the aid of object detection algorithms [60]. Robots are able to explore complex surroundings, operate objects, and carry out activities with precision and accuracy by identifying and recognizing entities of interest. In a nighttime haze environment, the resolution of captured photos can be substantially compromised due to many detrimental degradation causes [61]. Thus, the algorithms and methods for identifying things in photos must advance and become as effective as possible to be able to utilize the advanced hardware, even if camera and processor technology is advancing quickly and is more powerful than ever [62]. Recently, many researchers have focused on object detection based on CNNs; some of them include [63,64,65]. Figure 1 shows a basic framework for object detection or classification using CNNs.

### 4.4. Obstacle Avoidance

Combining many sensors, including cameras, radars, lidars, and ultrasonic equipment, to provide a complete and accurate picture of the environment is among the finest methods to employ robot perception and vision in obstacle avoidance. Using DL methods, including CNNs, to analyze sensor data and retrieve valuable features and patterns provides another method of using robotic perception and vision in obstacle avoidance [66]. Perception algorithms are now mainly task-specific and heavily reliant on human involvement and management. A vision-based framework for obstacle avoidance might be trained using CNNs, a classification-machine technique, and an actual surveillance picture. Thus, many researchers are working toward the development of advanced obstacle-avoidance algorithms based on CNNs for improving robotic perception [36,67].

## 5. Convolutional Neural Network-Based Robotic Perception

The problem of long-term localization and mobility in unfamiliar environments has become more and more crucial for assistance robots. These robots need to be able to handle mistakes in perception and must operate irrespective of unpredictable and unplanned environments. CNNs and Transformers are frequently used in image restoration tasks in recent times [68]. Trained CNN frameworks for real-world applications can extract features from raw input data, and CNNs offer better characteristics for unsupervised learning and interpretation. Robotic perception uses CNNs for a wide range of tasks, including detecting and localizing objects. Numerous automation activities, including navigation, object identification, quality assurance, and more, rely on computer vision, also known as machine vision in robotics, for perception. Computer vision-enabled robotic arms are frequently utilized production line activities to increase productivity. To understand and acquire data from images and videos, CNNs, as well as DL methods like Deep Reinforcement Learning (DRL) and Self-Organizing Maps (SOMs), are employed [69]. Robotics is evolving thanks to computer vision, which is also enhancing industries including manufacturing, agriculture, and healthcare.

Robotic perception refers to a robot’s capacity to analyze and understand its environment sufficiently to provide navigation and interaction within that context. At the heart of robot perception is the challenge of constructing an internal representation of the robot’s environment using integrated sensor data and existing information [70]. The built-in visualization of the environment might be solely geometric (including a point cloud), as seen in conventional simultaneous localization and mapping (SLAM), or it might include more advanced structures, including objects and other semantic components of the environment (including roads, buildings, and pedestrians). In this context, robot perception pertains to the subject of scene comprehension within the realm of computer vision studies. Robot perception has been a focal point of robotics studies for over 50 years, owing to its essential function in facilitating applications including navigation, path planning, and human-robot interaction. Perception has also been a central theme in the field of computer vision since it first emerged.

**Figure 10:**
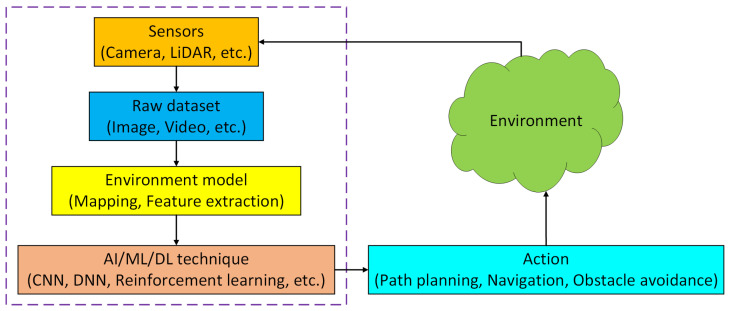
Illustration of a robotic perception framework.

As shown in Figure 10, the fundamental elements of a robotic perception framework comprise machine learning-based techniques, visualization of data (environment simulation), sensory data analysis, and decisions on taking actions. An essential component of a robotic perception framework is sensor-based environmental interpretation and mapping. Environment/scene depiction is synonymous with mapping in this context, which includes both obtaining an analytical framework and its conceptual explanation. The semantic mapping method employs DL at several levels, including determining, characterizing, and ideally fitting local regions based on multiple models or reasoning using spatial space and occlusions, i.e., along with more complex analyses [71]. However, the main function of environmental modeling in most applications is to represent input from sensory systems that are installed on the robot. This allows for interpretation and reasoning concerning the real environment that exists as the robotic system functions. Table 1 illustrates some major computer vision approaches using CNN-based image processing tasks on the basis of benchmark datasets UCF101 and HMDB51.

## 6. Future Research Trends and Challenges

Robotic perception represents a significant and emerging domain within robotics research. Perception research has the potential to significantly and immediately influence society at this moment. However, it is also simple to see that there continues to be an immense gap between the scope and resilience required of today’s robotic perception skills by several real-world applications of robotics and these skills themselves. Notwithstanding advancements in robotic perception, contemporary algorithms frequently exhibit fragility and fail unexpectedly when utilized beyond their training area (out of distributions) or when confronted with infrequent occurrences (the “tails” of the distributions) [79]. It is preferable to possess perception algorithms that readily adapt across many contexts, exhibit a well-defined operational domain, and can determine user trust during execution.

The execution of learning-based modules in contemporary perception systems mostly depends on graphics processing units (GPUs). Such systems’ substantial power demands frequently conflict with the constrained dimensions and performance of a robot. Even where there is sufficient room for complex processors (for instance, in self-driving automobiles), concerns are already raised by the associated consumption of power and environmental effects [80]. How to close this deficit is going to be a huge problem. New hardware and algorithms (such as application-specific integrated circuits for quick, energy-efficient computer vision) will probably need to be co-designed for this. On a deeper level, this additionally necessitates a more thorough examination of the relationships between embodiment, perception, and the downstream functions that perception must fulfill. Many datasets are developed for CNN-based robotics perceptions, but these datasets are not even enough for complete environment exploration. Due to insufficient information about the environment, it is important to be more focused on further studies to analyze weather conditions, unseen terrains, and variable lighting scenarios.

Developing multimodal algorithms and techniques that can reason about object interactions, perform sophisticated causal reasoning regarding the geometry, linguistics, and kinematics of the scene, and create cohesive representations of the environment is a major issue for robot perception. In particular, recent advancements in self-supervised pretrained vision-language foundation models (VLMs) have demonstrated remarkable efficacy in addressing challenging image processing and natural language processing issues [81]. These models make it possible to train on large amounts of unlabeled data from the Internet and do away with the requirement for specialized training datasets, but they also come with a higher level of computation that is barely manageable for robotic systems today. Creating lightweight VLMs that are simple to install and adaptable on different robots, integrating cutting-edge sensing modalities, and improving their interpretability and dependability for robotic activities remain difficult problems.

Regeneration and perception improvement approaches are current research fields in robotics that play critical roles in how we view and comprehend the environment. DL using computer vision is being significantly enhanced and developed, making it one of the biggest and most significant emerging topics in robotics. Object identification, categorization, classification, layout, network, performance, navigation, and exploration using spatial features are all expected to be key and profitable areas of research in robotics and AI in the upcoming years [82]. CNNs possess numerous shortcomings, including spatial information decline. Therefore, novel structures are necessary to address these issues.

Although CNNs have performed admirably in field experiments, there are still many problems that need to be looked at further. First of all, the deepening of modern CNNs demands tremendous computing power and large datasets for training. Collecting assigned datasets by hand takes a great deal of human labor. Therefore, it is desirable to investigate CNN unsupervised learning. Even if there are currently various asynchronous stochastic gradient descent (SGD) algorithms that use both GPU and central processing unit (CPU) groups to speed up the training process, it is still worthwhile to create efficient and adaptable concurrent training algorithms. These advanced models need significant amount of data and time during testing, which prevents them from being used on mobile systems with constrained resources. Researching ways to simplify and provide quick-to-run models without compromising accuracy is essential.

Secondly, a significant obstacle to using CNNs on a novel job is the need for significant expertise and experience in choosing appropriate hyperparameters, including the number of layers, learning rate, and kernel size. Due to their fundamental reliance, these hyperparameter values are very costly to tune. Current optimization methods for training deep CNN structures offer significant room for improvement, according to recent studies [83,84]. Lastly, there is currently a lack of a sound hypothesis regarding CNNs. The current CNN model performs excellently in a number of domains. However, in essence, we have no understanding of why or how it operates. It is preferable to invest more effort into researching the core ideas behind CNNs. Meanwhile, it is important to investigate how to use basic visual perception mechanisms to enhance the architecture of CNNs for robotic perception.

CNN-based computer vision and robotic perceptions stem from challenges in adaptability, generalization, and scalability in complex and real-world environments. Real-time perceptions for robotics are still challenging for the larger application of CNN models in perception. Sometimes, the CNN model fails to perform well in dynamic and evolving environments, which is the most important aspect for autonomous robots. Given that CNNs are not physical or visual operational simulations, it might prove difficult to understand the decisions they make. Uncertainty limits implementation and trust in safety-sensitive applications of robots, such as self-driving and medical services. CNNs are used by robotic surveillance devices for processing visual scenes in monitoring tasks, which can raise privacy concerns because some sensitive images can be processed during surveillance.

## 7. Conclusions

This paper provides a compact review with very important information about CNNs. CNNs are frequently used in studies and business due to their benefits, including regional connection, weight collaboration, and reducing dimensionality via downsizing. This article presents a comprehensive overview of CNNs, including its basic principles, convolution and pooling methods, various types of CNNs, applications, and potential prospects. Additionally, we discussed the fully connected layers, which are an essential part of CNNs. This article provides outstanding information about the application of CNNs in robotic perception by using several image processing techniques, including HAR, human pose estimation, and object detection. Robotic perception using CNNs is extensively described in this paper. Furthermore, we discussed the obstacle avoidance techniques that can be used for robotic perception. This paper will play an important role in providing basic information to young researchers about CNNs as well as important information about its role in robotic perception.

## Figures and Tables

**Table 1:** Accuracy comparison using state-of-the-art methods on the UCF101 and HMDB51 datasets.

Technique	Type of CNN	Type of Input	Benchmark Dataset (Accuracy in %)	
			UCF101	HMDB51
Sequential Segment Networks (SSN) [72]	Batch Normalized Inception (BN-inception)	RGB	94.80	73.80
Multiplier Two-stream [73]	RESNet-50	RGB	94.20	68.90
KeyVolume [74]	GoogLeNet	RGB	93.10	63.30
Temporal Scale-Invariant [75]	VGG-16	RGB	93.70	69.50
Long-term Temporal Convolutions (LTC) [76]	LTC	RGB	92.70	67.20
Trajectory-pooled Deep-convolutional Descriptor (TDD) [77]	ZFNet	RGB	91.50	65.90
Temporal Segment Network (TSN) [78]	ResNet	RGB	94.90	71.00

## Data Availability

Not applicable.
